# Furosemide use in Italian neonatal intensive care units: a national survey

**DOI:** 10.1186/s13052-020-00851-2

**Published:** 2020-06-22

**Authors:** Valeria Anna Manfredini, Chiara Cerini, Antonio Clavenna, Andrea Dotta, Maria Letizia Caccamo, Alex Staffler, Luca Massenzi, Rossano Massimo Rezzonico, on the behalf of study Group of Neonatal Pharmacoterapy of the Italian Society of Neonatology

**Affiliations:** 1 Neonatal Intensive Care Unit, ASST Rhodense, Via C. Forlanini 95, 20024 Milan, Italy; 2 Children’s Hospital Los Angeles, 4650 Sunset Boulevard, Los Angeles, CA 90027 USA; 3 Department of Public Health, Laboratory for Mother and Child Health, Istituto di Ricerche Farmacologiche Mario Negri IRCCS, Milan, Italy; 4 Department of Medical and Surgical Neonatology, Neonatal Intensive Care Unit, Bambino Gesù Children Hospital, IRCCS, Rome, Italy; 5 Indipedent researcher, Former director of Neonatal Intensive Care Unit, Sant’Anna Hospital, Como, Italy; 6 Division of Neonatology, Central teaching Hospital of Bolzano, Bozen, Italy; 7 Neonatology and Neonatal Intensive Care Unit, “San Giovanni Calibita” Fatebenefratelli Hospital, Rome, Italy; 8 Indipendent researcher, Former director of Neonatal Intensive Care Unit, ASST Rhodense, Via C. Forlanini 95, 20024 Milan, Italy

**Keywords:** Furosemide, Newborn, Posology, Off-label

## Abstract

**Abstract:**

**Background:**

Furosemide is approved in full term neonates to treat edema associated with congestive heart failure, cirrhosis and renal diseases. It is often administered off-label in premature neonates, to treat respiratory conditions and at doses greater-than-recommended. We conducted a national survey on behalf of the Neonatal Pharmacotherapy Study Group of the Italian Society of Neonatology (SIN), to investigate its use in Italian neonatal intensive care units (NICUs), in conformity with current guidelines.

**Methods:**

Between December 2016 and June 2017, a 14-item multiple-choice online questionnaire was sent to all NICU directors from the SIN directory.

*G*estational age, route of administration, posology, indications, referenced guidelines, adverse effects monitoring and the presence of Paediatric Cardiology or Cardiosurgery service on site were assessed. A chi-square test was performed 1) to evaluate differences in the distribution of responses between NICUs administering furosemide at doses higher-than-recommended; 2) to compare the proportion of NICUs administering furosemide at high doses in institutions with versus without a Paediatric Cardiology or Cardiosurgery service.

**Results:**

The response rate was 50% (57/114). The intravenous and oral routes were chosen primarily; the intravenous administration in single doses predominated over continuous infusion. Its main therapeutic indications were congestive heart failure/overload (94.7%) and oligo-anuria (87.7%) however furosemide was also frequently used for broncopulmonary dysplasia (50.9%) and respiratory distress syndrome and/or transient tachypnea of the newborn (24.6%).

In 28/57 NICUs furosemide was administered at doses higher-than-recommended. In most NICUs the same posology was used in term and preterm neonates. Compared to the total sample, a larger proportion of NICUs administering doses greater-than-recommended referenced current literature for reasons to do so (19.3 and 32.1% respectively). The presence of a Paediatric Cardiology or Cardiosurgery service on site did not correlate with the chosen posology.

The majority of NICUs performed acoustic test and renal ultrasound for furosemide exposure greater than 2 weeks.

**Conclusions:**

In Italian NICUs, furosemide is commonly prescribed to term and preterm newborns for label and unlabeled indications. Doses greater-than-recommended are frequently administered. Such use is not necessarily inappropriate. More research is required to assess the efficacy and safety of unlabeled use.

## Background

Furosemide is one of the most commonly prescribed drugs by Italian neonatologists and worldwide [1–3]. Its diuretic effect is exerted in the renal tubules and in the loop of Henle where it inhibits sodium and chloride reabsorption producing prompt and vigorous diuresis that usually lasts 4 to 6 h [4, 5].

In children and adults, furosemide is approved by the Food and Drug Administration (FDA) and by the Italian Medicines Agency (AIFA) for the treatment of edema associated with congestive heart failure, hepatic cirrhosis and nephrotic syndrome.

In Neonatal Intensive Care Units (NICUs) the administration of furosemide is extended to the off-label treatment of respiratory conditions of preterm and term neonates including bronchopulmonary dysplasia (BPD), respiratory distress syndrome (RDS), and the transitory tachypnea of the newborn (TTN) [6–9].

The use of furosemide in the treatment of respiratory diseases of the term and preterm newborn has been driven by the evidence of a diuresis-induced decrease in extracellular volume and increase in the oncotic pressure following its administration, that would reduce lung edema during the early stages of chronic lung disease and in the acute phases of both TTN and RDS [10–12].

For children and term infants, the usual initial dose of oral furosemide is 1–2 mg/kg body weight (AIFA) or 2 mg/kg (FDA). If the diuretic response is not satisfactory after the initial dose, the FDA allows increasing the dose by 1 or 2 mg/kg no sooner than 6 to 8 h after the previous dose. Maximum oral daily doses greater than 40 mg (AIFA) or 6 mg/kg (FDA) are not recommended.

Because of insufficient data in the pediatric population, the AIFA gives no advise for the intravenous (i.v.) posology.

Conversely, the FDA suggests that the usual initial dose of furosemide is 1 mg/kg body weight injected intramuscularly or intravenously with slow push under close medical supervision, and that it may be increased by 1 mg/kg until the desired diuretic effect is obtained, not sooner than 2 h after the previous dose. Daily i.v. doses greater than 6 mg/kg are not recommended.

Current guidelines from the Italian Society of Neonatology (SIN) state that in term infants, furosemide can be administered intravenously, orally or intramuscularly at the dose of 1 mg/kg/dose every 12 h and increased until the desired effect is obtained. No maximum dose is specified in the current SIN guidelines.

In preterm neonates (gestational age < 37 weeks), clinical trials of drug safety, dosing, and efficacy are lacking. However, the drug is employed by neonatologists to improve oxygenation and lung compliance in premature infants [7]. Moreover, in the United States it is administered to as many as 50% of hospitalized extremely low birth weight (ELBW) patients [13]. In Italy, the retrospective study by Girardi et al. in neonates < 37 weeks gestational age in a tertiary level NICU in Bologna (Northern Italy) showed that 25% of neonates with birth weight 1000–1500 g and 72% of neonates weighting < 1000 g were exposed to furosemide [1].

According to the FDA, for very preterm infants (gestational age less than 31 weeks) the maximum dose of i.v. furosemide should not exceed 1 mg/kg/day because of potential toxicity.

Similarly, the current SIN guidelines state that very preterm infants can receive furosemide 1 mg/kg every 24 h.

Regardless of gestational age, the SIN recommends the dose of 0.1–0.2 mg/kg/hour for continuous i.v. infusion, not to exceed 4 mg/min.

The most common side effects of furosemide exposure are excessive diuresis, dehydration, blood volume reduction with circulatory collapse, and electrolytes depletion.

In premature infants, furosemide may precipitate nephrocalcinosis/nephrolithiasis, for which renal function monitoring is warranted and sonography must be considered [14, 15].

A potential association between furosemide exposure and patent ductus arteriosus (PDA) poses further limitation to its use especially in preterm newborns, in which pulmonary overflow due to a hemodynamically significant PDA may benefit from diuretics administration [16]. With this background, we conducted a national survey on behalf of the Neonatal Pharmacotherapy Study Group of the SIN, to investigate the use of furosemide in Italian NICUs. Our primary aim was to investigate whether and how the use of furosemide differs in the every-day clinical practice in Italian NICUs with respect to the adherence to the current label instructions, guidelines and scientific evidence. Secondarily, we investigated how side effects were monitored.

## Methods

Between December 2016 and June 2017 all the Italian level III NICU directors listed in the SIN directory were asked via email to complete an online questionnaire created with Survey Monkey. The 14-item questionnaire was in multiple-choice format and required approximately 15 min to be filled out. The NICU directors were instructed to answer the questionnaire based on their institution’s policy. Where indicated, participants could select *more than one answer.* If a timely response was not obtained, investigators made a follow up phone call or sent a reminder email. Participants were asked to specify their institution to avoid duplicated data. All identities were kept confidential throughout data collection and analysis. The survey was administered in Italian and then translated in English for publication (Table 1).

**Table 1:** survey questions and frequency of selected answers

***N°***	***Questions***	***Answers***	***All centers***		***Centers prescribing higher doses***		p-value*
			***N = 57***	***%***	***N = 28***	***%***	
**1**	**Do you administer furosemide to newborns regardless of their gestation age?**	Yes	**55**	96.5	**26**	92.9	n.s.
		No	**2**	3.5	**2**	7.1	
**2**	**Which route do you use to administer furosemide? Multiple choices allowed**	Intravenous	**57**	100.0	**28**	100.0	n.s.
		Oral	**43**	75.4	**22**	78.6	
		Nebulized	**4**	7.0	**2**	7.1	
		Intramuscular	**3**	5.3	**1**	3.6	
**3**	**For which clinical indications do you use furosemide?** **Multiple choices allowed**	Cardiac overload	**54**	94.7	**28**	100.0	n.s.
		Oligo/anuria	**50**	87.7	**26**	92.9	
		BPD	**29**	50.9	**14**	50.0	
		RDS/TTN	**14**	24.6	**8**	28.6	
		Other	**2**	3.5	**1**	3.6	
**4**	**Which intravenous administration do you use?** **Multiple choices allowed**	Single doses, eventually repeated	**49**	86.0	**23**	82.1	n.s.
		Continuous infusion	**24**	42.1	**12**	42.9	
		Single doses plus continuous infusion	**18**	31.6	**8**	28.6	
**5**	**At which maximum dose do you administer furosemide intravenously (single dose)?**	1–2 mg/kg	**35**	61.4	**6**	21.4	n.a.
		> 2–4 mg/kg	**17**	29.8	**17**	60.7	
		> 4–10 mg/kg	**4**	7.0	**4**	14.3	
		> 10 mg/kg	**1**	1.8	**1**	3.6	
**6**	**In full term newborns, how often do you administer furosemide intravenously?**	Every 12 h or more	**24**	42.1	**8**	28.6	n.s.
		Accordingly to diuresis	**21**	36.8	**12**	42.9	
		Every 8 to 12 h	**6**	10.5	**5**	17.9	
		Every less than 8 h	**5**	8.8	**3**	10.7	
**7**	**When repeated intravenous doses are needed, which is the minimum time interval between single doses?**	At least 6 h	**29**	50.8	**12**	42.9	n.s.
		At least 4 h	**17**	29.8	**8**	28.6	
		At least 1 h	**9**	15.7	**8**	28.6	
		Not applicable	**2**	3.5	**1**	3.6	
**8**	**In full term newborns, which dose do you use for intravenous continuous infusion? Multiple choices allowed**	0.1–0.2 mg/kg/h	**31**	54.4	**10**	35.7	n.a.
		0.2–0.4 mg/kg/h	**11**	19.3	**9**	32.1	
		0.4–1 mg/kg/h	**6**	10.5	**5**	17.9	
		1 mg/kg/h	**2**	3.5	**2**	7.1	
		Non applicabile	**15**	26.3	**7**	25.0	
**9**	**In preterm newborns less then 32 weeks gestational age, do you use different posology? Multiple choices allowed**	No	**33**	57.9	**12**	42.9	0.045**
		Yes, smaller doses	**15**	26.3	**11**	39.3	
		Yes, longer intervals	**11**	19.3	**6**	21.4	
		Yes, we greater doses	**2**	3.5	**1**	3.6	
		Yes, shorter intervals	**0**	0.0	**0**	0.0	
**10**	**In general, do you use different posology for different clinical indication?**	Yes	**39**	68.4	**21**	75.0	n.s.
		No	**18**	31.6	**7**	25.0	
**11**	**For which clinical indications do you use maximum doses higher than 1–2 mg/kg or 0.1–0.2 mg/kg/hour? Multiple choices allowed**	Cardiac overload	**20**	35	**20**	71.4	n.a.
		Oligo/anuria	**11**	19.3	**11**	39.2	
		RDS/TTN	**1**	1.8	**1**	3,5	
		Not applicable	**29**	50.9	**0**	0.0	
		Other	**2**	3.5	**2**	7.1	
**12**	**Which protocol/guideline/prescribing indication do you refer to for the appropriate dosage and administration in different scenarios? Multiple choices allowed**	Manuals of neonatology or neonatal pharmacotherapy	**48**	84.2	**24**	85.7	0.006
		Articles from the literature	**11**	19.3	**9**	32.1	
		Center protocols	**9**	15.8	**6**	21.4	
		No specific guideline/indication	**7**	12.3	**0**	0.0	
**13**	**Have you ever had to discontinue the treatment because of drug related adverse events?** **If yes, which adverse events did occur?**	No	**43**	75.4	**22**	78.6	n.s.
		Yes	**14**	24.6	**6**	21.4	
		Metabolic alcalosis	**5**	8.8	**2**	7.1	
		Dyselectrolytemia	**5**	8.8	**2**	7.1	
		Nephrocalcinosis	**5**	8.8	**3**	10.7	
		Hypotension	**4**	7.0	**1**	3.6	
**14**	**In case of prolonged treatment (> 2 weeks), how do you monitor for side effects?**	Renal ultrasound	**4**	7	**3**	10.7	n.a.
		Acoustic test	**4**	7	**3**	10.7	
		Both	**27**	47.4	**18**	64.3	
		No follow up	**4**	7	**0**	0	
		Not applicable (length of treatment < 2 weeks)	**18**	31.6	**4**	14.3	

* Chi-square test comparing NICUs with high dose of furosemide versus standard dose; ** No versus Yes (different posology used in preterm)

*n.s.* not significant; *n.a.* not applicable

*The questionnaire addressed* gestational age criteria for the administration of furosemide, route of administration, dosing intervals, treatment indications and mode of administration (e.g. continuous infusion versus single dose). Additional questions aimed to investigate which guidelines or references were used, how adverse effects were monitored and the presence of Paediatric Cardiology and Paediatric Surgery services on site. Surveys data were collected on an Excel database.

The study is mainly descriptive. Data are reported as number and percentage of responders.

A chi-square test was performed 1) to evaluate differences in the distribution of responses between NICUs administering furosemide at doses higher-than-recommended by neonatal guidelines (i.e. > 1–2 mg/kg for the i.v. single dose or 0.1–0.2 mg/kg/h for the i.v. continuous infusion); 1) to compare the proportion of NICUs administering furosemide at high doses in institutions with versus without a Paediatric Cardiology or Cardiosurgery service.

## Results

A total of 114 eligible NICUs were identified via the SIN directory. The overall response rate was 50% (57/114). Twelve NICUs had a Paediatric Cardiology or Cardiosurgery service (data not shown). Frequency distribution of responses to survey question is shown in Table 1.

In almost all NICUs (*n* = 55; 96.5%) furosemide was prescribed regardless of the newborn's gestational age.

Congestive heart failure/overload and oligo-anuria were reported as the main therapeutic indications (94.7 and 87.7%), while respiratory conditions including BPD and RDS/TTN were selected by 50,9 and 24.6% of the surveyed centers, respectively.

Intravenous (*n* = 57; 100%) and oral (*n* = 43, 75.4%) were the most common routes of administration. Regarding the i.v. route, furosemide was administered in single doses versus continuous infusion in 49 (86%) versus 24 (42.1%) NICUs; in 18 NICUs (31.6%) the drug was administered in single doses additional to continuous infusion.

In half of the NICUs (28/57) furosemide was administered at doses higher-than-recommended by neonatal guidelines. Cardiac overload and oligo-anuria were confirmed as the most frequent indications for which such high doses were used.

Restricting the analysis to the 28 NICUs in which furosemide was administered at higher-than-recommended doses, diuretic response prevailed as the guiding criterium for the repetition of single doses (12/28); moreover, a shortened 1-h interval between doses was reported in higher proportion (28.6%) compared to the total sample (15.7%).

A lower gestational age limit for administration of furosemide was indicated by two centers only (3.5%) as 25 and 27 weeks gestational age respectively (data not shown). Most institutions (57.9%) reported the same posology in term and preterm infants. When restricting the analysis to NICUs administering furosemide at high doses, in 39.3% the drug was given to very preterm babies in smaller doses than those used for term infants, while 21.4% surveyed centers administered furosemide to very preterm babies with longer intervals between doses as compared to term infants.

The great majority of NICUs (84.2%) referenced to manuals of neonatology or neonatal pharmacotherapy for posology, while no specific guidelines or indications were followed in 12.3% institutions.

The presence of a Paediatric Cardiology or Cardiosurgery service (data not shown) was not related to the use of higher doses (χ2 test 0.15 – *p* = 0.7).

Of note, institutions administering doses higher-than-recommended referenced the literature for posology in a significantly higher proportion compared to the total sample (32.1% versus 19.3%; χ2 test 4.3– *p* = 0.006).

Fourteen NICUs (24.6%) reported treatment discontinuation due to one or more adverse events including metabolic alkalosis (*n* = 5), dyselectrolytemia (*n* = 5), nephrocalcinosis (*n* = 5) and hypotension (*n* = 4). No relationship was found between treatment discontinuation and high dosing.

A complete screening including acoustic test and renal ultrasound was reported by the majority of respondents (27/39, 69.2%) and the proportion increased when restricting the analysis to NICUs prescribing high doses (18/24, 75%). A policy for side effect monitoring of patients receiving prolonged treatment (> 2 weeks) was not available in four centers (7%).

## Discussion

Furosemide is the diuretic most commonly used in infants [1–3]. Whereas it is approved for the treatment of edema associated with congestive heart failure, hepatic cirrhosis and nephritic syndrome, it is also extensively used in term and premature neonates with respiratory conditions such as TTN, RDS and BPD.

Indeed, highly variable responses to furosemide administered in standard doses of 1–2 mg/kg are observed in newborns, suggesting that such doses might not induce the desired diuretic response [17]. This is partly due to the immature kidney function of the newborn; in addition, the diuretic response depends on the disease, the severity of the clinical presentation, and the route of administration [5].

Moreover, the bioavailability of oral furosemide in infants appears to be low and the literature mostly provides data on intravenous administration [18].

Given the great variability in response to furosemide and the flexible use of the drug in the neonatal population, both in terms of indication and posology, we investigated the common practice for administration in Italian NICUs, including the off-label use in preterm infants.

To the best of our knowledge, this is the first study investigating the use of furosemide use in this setting.

Our survey confirms an extensive use of furosemide in Italian NICUs. Moreover, it is administered at doses higher than those described by the FDA, AIFA or SIN, both in term and preterm infants. In addition, furosemide is extensively used for the treatment of respiratory conditions.

Three Cochrane systematic reviews have shown, however, inconsistent results and failed to find clear advantages using furosemide to treat any of the aforementioned respiratory conditions [7–9]. These results mainly derived from studies in the pre-ncpap era and testing doses of furosemide < 2 mg/kg every 12 or 24 h. Recently, an observational study including more than 800 extremely preterm infants (23 0/7–28 6/7 weeks gestational age) failed to prove any association between diuretics use and short-term improvement in respiratory support [6].

Considering BPD, a recent retrospective analysis including more than 36,000 ELBW premature infants showed that furosemide exposure per se is not related to a decrease of either BPD nor the combined outcome of BPD or death. However, a significant decrease in BPD and composite outcome BPD or death was observed with a 10-percentage points increase in the proportion of days exposed to the drug [19].

The presence of a Paediatric Cardiology or Cardiosurgery service was assessed as a factor *potentially* influencing furosemide administration, in particular the chosen dose. Surprisingly, no correlation emerged between the presence of the above services and the prolonged (> 2 weeks) or off-label administration of furosemide.

Notably, systematic consultation of manuals of neonatology or neonatal pharmacotherapy for posology was reported in most institutions. However, in many NICUs (38.6%) furosemide was administered in i.v. single doses higher than 1–2 mg/kg/d.

In critically ill patients, furosemide has been administered in high dosage regimens on the presumption that this practice is safe in the setting of a rapid diuretic response with tight monitoring of fluid balance. Several authors have shown that high dosage regimens are indeed safe and well tolerated in critically ill neonates [17, 20–22].

We infer that the off-label use recorded in our survey is due to expected advantages or failure of standard treatments and further supported by the absence of acute adverse events.

Interestingly, when considering only those institutions administering furosemide in single i.v. doses higher-than-recommended, we observed a significantly higher number of NICUs referencing to articles from the literature. Such results suggest that therapeutic choices differing from what is suggested by common neonatal therapeutic guidelines are mainly taken according and supported by the existing scientific evidence.

We found that the administration of furosemide in single doses was twice as frequent as the continuous infusion. Moreover, we observed a strong tendency to administer higher daily amount via repeated single doses (82.1%) compared to continuous infusion (42.9%).

Remarkably, these findings are not consistent with the current evidence that continuous administration of furosemide in postoperative pediatric patients leads to more controlled diuresis, greater urinary output per dose and smaller cumulative amount of drug [20, 21, 23, 24].

In infants, furosemide administered i.v. in single doses of 1 mg/kg/dose leads to a very steep logarithmic dose-response curve which may suggest that single high doses could not determine a significant increase in diuretic response [17]. The time of furosemide delivery to the receptor in the kidney seems to improve with continuous infusion, providing a more important determinant of the diuretic response than the total amount of drug administered [25, 26].

More studies, outside the critical setting of cardiac surgery, are needed to test whether continuous infusion of furosemide is more advantageous for NICU patients as well [17].

In premature infants adverse events are a major concern to the use of unlabeled medications. Furosemide exposure in preterm infants is a strong risk factor for ototoxicity and sensorineural hearing loss (SNHL) [27–30], and has been implicated in the development of nephrocalcinosis and nephrolithiasis (NC/NL) [14, 15, 31]. There is evidence that the risk for ototoxicity is higher with the i.v. compared to the oral administration [18], with prolonged treatment, high cumulative exposure [29], and for blood levels exceeding 50 μg/mL [32]. Similarly, renal calcifications are documented in patients receiving doses of > 2 mg/kg/d for at least 12 days [31].

Close monitoring of the side effects is thus mandatory. In our survey, surveillance for adverse events after prolonged exposure was performed via renal ultrasound and/or acoustic test by the vast majority of prescribing centers, for standard (61.4%) as well as for higher doses (85,7%). Despite the need for implementation of monitoring is undoubted, it seems that the use of higher doses and/or prolonged treatments is followed by a slightly more accurate follow-up.

It is well known that the volume of distribution, clearance, and half-life of the drug may change in preterm infants depending on gestational age, birth weight and postnatal age [18, 33–35].

Remarkably, in our study most NICUs reported the same posology in term and preterm infants and in only two institutions furosemide was not administered to infants with gestational age less than 25 or 27 weeks.

This is in line with previous research, in which furosemide treatment was extended to patients of extremely low birth weight and gestational age [13, 19]. We did not assess if any institutions used the birth weight as limiting criteria for furosemide administration.

Additional risks are associated to the length of treatment. Infants less than 32 weeks gestational age are the most vulnerable given their kidney immaturity [33]. Previous research showed that preterm infants are commonly exposed to prolonged treatment with average exposure duration of at least 12 days [1, 36]. Furthermore, recent data show that preterm newborns exposed to furosemide suffer from more severe comorbidities than unexposed patients [6, 19, 36]. Indeed, long-term exposure to furosemide (greater than 14 days) in extremely preterm infants warrants even closer monitoring of side effects.

Adverse events requiring treatment discontinuation were reported by less than one third (14/57) of the surveyed centers. These included metabolic alkalosis, dyselectrolytemia, nephrocalcinosis and hypotension that are described in literature as related to furosemide administration, mainly due to long-term exposure [5]. Our survey was not designed to specifically collect data regarding treatment discontinuation. Thus, we are not able to find a precise correlation between baseline clinical presentation, dosing schedule and adverse events. Regardless, no relationship between the administration of high doses or prolonged treatment and the need to discontinue the therapy was evident. The low rate of adverse event is in line with a recent review that found low evidence for the risk of SNHL and NC/NL in premature infants [37].

The main limits of our study were the small sample size and the fair response rate of 50%, which is lower than the standard for survey research and has determined loss of statistical power [38]. Because of the study’s exploratory nature, however, the representation wasn’t as important as in descriptive surveys and lower response rates did not impact the research outcome.

Worldwide, little consensus has been reached on the off-label use of this drug, and therefore expert interpretation of current evidence and future research should be prioritized. New investigator-initiated research is required to assess the safety of furosemide in premature infants when its use is extended to the treatment of respiratory conditions such as BPD, RDS and TTN.

The results from our study may suggest that higher doses prove advantageous in managing certain clinical scenarios of the preterm newborn pathology. However, in order to promote the rational use of medicines and to achieve positive and safe patient outcomes, clinical data should be collected regarding the use of doses higher-than-recommended. Further work needs to be done to decide whether the original doses recommended for use in critically ill newborns are valid.

A phase II randomized controlled trial by the Paediatric Trials Network (PTN) is ongoing to test the safety of furosemide in preterm newborns < 29 weeks gestational age at risk of BPD receiving positive airway pressure or mechanical ventilation [39].

To the best of our knowledge, no other trials are ongoing or about to be published to test the safety and efficacy of higher doses of furosemide in preterm newborns.

## Conclusions

In Italian NICUs, furosemide is commonly administered to term and preterm infants for label and unlabeled indications including respiratory conditions. Doses higher-than-recommended are frequently administered, mainly for cardiac overload and oligo-anuria. Yet little is known about the drugs’ potential side effects and adverse events especially in preterm and very preterm neonates. Off-label drug use is widely reported, although little to no information is available regarding efficacy and safety [40–43]. Adequate drug use in neonatal care is mostly derived from the available evidence and the medical experience, with wide variations in local policies and newborn characteristics and does not necessarily imply inappropriate drug use.

Undoubtedly, systematic monitoring of side effects is mandatory and data concerning outcomes should be collected.

## Data Availability

Data were used under license of the current study by the Italian Society of Neonatology and are not publicly available. However, data are available from the corresponding author upon reasonable request and with the permission of the Italian Society of Neonatology (group of newborn pharmacotherapy).

## Abbreviations

FDA: Food And Drug Administration
AIFA: Italian Medicines Agency
NICUs: Neonatal intensive care units
BPD: Bronchopulmonary dysplasia
RDS: Respiratory distress syndrome
TTN: Transitory tachypnea of the newborn
IV: Intravenous
SIN: Italian society of neonatology
PDA: Patent ductus arteriosus
SNHL: Sensorineural hearing loss
NC: Nephrocalcinosis
NL: Nephrolithiasis
